# LCAT, ApoD, and ApoA1 Expression and Review of Cholesterol Deposition in the Cornea

**DOI:** 10.3390/biom9120785

**Published:** 2019-11-26

**Authors:** Rhonda Flores, Xueting Jin, Janet Chang, Connie Zhang, David G. Cogan, Ernst J. Schaefer, Howard S. Kruth

**Affiliations:** 1 Experimental Atherosclerosis Section, National Heart, Lung, and Blood Institute, National Institutes of Health, Bethesda, MD 20892, USA; rhonda.flores@gmail.com (R.F.); tina.jin@nih.gov (X.J.); changj@nhlbi.nih.gov (J.C.); 2 Advanced Cell Diagnostics, Newark, CA 94560, USA; Connie.Zhang@bio-techne.com; 3 Cornea and Cataract Section, National Eye Institute, National Institutes of Health, Bethesda, MD 20892, USA; kruthh@nih.gov; 4 Cardiovascular Nutrition Laboratory, Human Nutrition Research Center on Aging at Tufts University and Tufts University School of Medicine, Boston, MA 02111, USA; eschaefer@BostonHeartDx.com; 5 Boston Heart Diagnostics, Framingham, MA 01702, USA

**Keywords:** cornea, keratocytes, LCAT, ApoA1, ApoD, cholesterol, HDL, atherosclerosis, corneal stromal fibroblasts

## Abstract

Lecithin:cholesterol acyltransferase (LCAT) is an enzyme secreted by the liver and circulates with high-density lipoprotein (HDL) in the blood. The enzyme esterifies plasma cholesterol and increases the capacity of HDL to carry and potentially remove cholesterol from tissues. Cholesterol accumulates within the extracellular connective tissue matrix of the cornea stroma in individuals with genetic deficiency of LCAT. LCAT can be activated by apolipoproteins (Apo) including ApoD and ApoA1. ApoA1 also mediates cellular synthesis of HDL. This study examined the expression of LCAT by epithelial cells, keratocytes, and endothelial cells, the cell types that comprise from anterior to posterior the three layers of the cornea. LCAT and ApoD were immunolocalized to all three cell types within the cornea, while ApoA1 was immunolocalized to keratocytes and endothelium but not epithelium. In situ hybridization was used to detect LCAT, ApoD, and ApoA1 mRNA to learn what cell types within the cornea synthesize these proteins. No corneal cells showed mRNA for ApoA1. Keratocytes and endothelium both showed ApoD mRNA, but epithelium did not. Epithelium and endothelium both showed LCAT mRNA, but despite the presence of LCAT protein in keratocytes, keratocytes did not show LCAT mRNA. RNA sequencing analysis of serum-cultured dedifferentiated keratocytes (commonly referred to as corneal stromal fibroblasts) revealed the presence of both LCAT and ApoD (but not ApoA1) mRNA, which was accompanied by their respective proteins detected by immunolabeling of the cultured keratocytes and Western blot analysis of keratocyte lysates. The results indicate that keratocytes in vivo show both ApoA1 and LCAT proteins, but do not synthesize these proteins. Rather, keratocytes in vivo must take up ApoA1 and LCAT from the corneal interstitial tissue fluid.

## 1. Introduction

**Figure 1:**
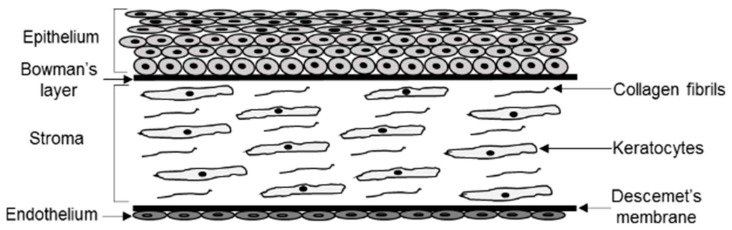
Cross sectional schematic representation of the human cornea. The cornea is an avascular tissue comprised of five layers. The outermost anterior layer is the epithelial layer that lies adjacent to the acellular Bowman’s connective tissue layer. This is followed by the stroma, which is the thickest layer of the cornea and consists of keratocytes embedded in dense bands of collagen fibrils. Acellular Descemet’s membrane separates the stroma from the most posterior layer, which consists of a monolayer of endothelial cells.

The corneal stroma is a dense connective tissue composed of collagen and proteoglycans with embedded cells called keratocytes (Figure 1). The corneal stroma is covered by a multilayer of corneal epithelium facing outward that is bathed by tear fluid. The stroma is covered by an inward facing monolayer of corneal endothelial cells (not to be confused with vascular endothelium) that contacts the aqueous fluid. Clarity of the cornea is essential to maintain visual acuity. In this regard it is interesting that genetic diseases with defects in lipid clearing mechanisms result in cholesterol deposition within the corneal stroma. In some cases, the degree of cornea clouding caused by lipid deposition is such that a corneal transplant is required to restore normal vision. Genetic mutations in *ABCA1* (Tangier disease), *LCAT* (familial lecithin:cholesterol acyltransferase (LCAT) deficiency, which exhibits renal disease and anemia, and fish-eye disease, which does not exhibit renal disease or anemia), *ApoA1* (familial apolipoprotein (Apo) A1 deficiency), and *UBIAD1* (Schnyder corneal dystrophy) all produce varying degrees of corneal cloudiness due to corneal accumulation of lipid including cholesterol [1,2,3]. The first three diseases are associated with abnormalities in so called reverse cholesterol transport from tissues and are accompanied by deficient and abnormal high-density lipoprotein (HDL) particle distributions [4].

Research has shown that mobilization of cholesterol from cells and tissues involves a stepwise process in which a special class of amphipathic apolipoproteins, most importantly ApoA1 and ApoE, interacts with an ATP-binding cassette transporter protein, ABCA1, that results in complexing of phospholipid with the amphipathic apolipoprotein, forming a discoidal structure [5,6,7]. This discoidal complex, often referred to as nascent high-density lipoprotein (HDL), functions to solubilize excess cholesterol present either within the cell plasma membrane [8] or cholesterol that cells shed into the extracellular matrix [9]. The capacity of the discoidal apolipoprotein–phospholipid complex to solubilize cholesterol is enhanced by the action of lecithin:cholesterol acyltransferase (LCAT) [10], an enzyme secreted by the liver into the plasma [11,12,13]. LCAT esterifies cholesterol within the complex by transferring an acyl group from the sn2-position of phosphatidylcholine (i.e., lecithin) to the 3-hydroxyl group of cholesterol. The formed cholesteryl ester oil moves into and expands the core region of nascent HDL converting the nascent discoidal-shaped HDL into a mature spherical-shaped HDL particle [14]. With discoidal HDL, LCAT function is enhanced primarily by apolipoprotein (Apo)A1, and with mature spherical HDL further enhanced by other amphipathic apolipoproteins including ApoD and ApoE [15,16]. Furthermore, addition of ApoE to HDL provides for further enlargement of HDL size and increases its cholesterol carrying capacity [17]. Although potentially involved in many steps of reverse cholesterol transport, ApoE function does not appear to be rate limiting for cholesterol removal from the cornea because, in contrast to genetic deficiency of ABCA1, LCAT, and ApoA1, genetic deficiency of ApoE has not been reported to cause lipid accumulation in the cornea [18].

Given the importance of maintaining clarity of the cornea, it is of interest to understand how the cornea maintains lipid homeostasis and mobilizes excess cholesterol. The corneal stroma where cholesterol accumulates in the above-mentioned genetic diseases is very similar in structure to the artery wall where abnormal accumulation of cholesterol causes atherosclerosis. Both the cornea and artery wall are dense connective tissues embedded with keratocytes and smooth muscle cells, respectively. Learning about corneal regulation of cholesterol homeostasis could provide insight into how the vessel wall maintains cholesterol homeostasis, or in the case of atherosclerosis fails to prevent cholesterol accumulation. 

Deposition of lipid in the cornea is not limited to just genetic diseases of HDL metabolism; the peripheral cornea can be a site of lipid deposition producing a distinct partial arc or complete circle of lipid referred to as arcus lipoides. Besides occurring in deficiencies or defects in LCAT, ApoA1, and UBIAD1, peripheral arcus occurs with aging showing an incidence of close to 50% in males past the age of 40 [19]. A peripheral arcus can occur as early as the first decade of life when levels of low density lipoproteins (LDL) are elevated such as occurs in familial hypercholesterolemia [20]. Extracellular LDL and LDL-derived cholesteryl ester-rich small lipid droplets (≈100 nm diameter) like those that accumulate in human atherosclerotic lesions give rise to the peripheral arcus [21,22,23]. LDL, HDL, and other plasma components are delivered to the cornea via the limbal vasculature that encircles the peripheral cornea [24]. LDL is small enough to diffuse from the limbal vasculature into the peripheral cornea. However, in contrast to HDL (<10 nm), LDL is too large (22 nm) to diffuse into the central cornea [24,25]. Lipid deposition in the peripheral cornea does not interfere with vision, but as described above lipid deposition in the central cornea does interfere with vision. 

To better understand abnormal cholesterol accumulation within the cornea, we have investigated the presence and cellular expression of LCAT and two of its activators, ApoD and ApoA1, [26], in the cornea and in keratocytes cultured from the cornea. In addition, we review the possible sources of cholesterol that accumulates and possible mechanisms involved in its removal. 

## 2. Materials and Methods

### 2.1. Western Blot Assay

Human keratocytes (ScienCell #6520, Carlsbad, CA, USA) and human primary dermal fibroblasts (ATCC #PCS-201-012, Manassas, VA, USA) were cultured in fibroblast medium (ScienCell #2301) containing 2% fetal bovine serum (FBS), fibroblast growth supplement in an amount specified by the manufacturer (ScienCell #2352,), 50 units/mL of penicillin, and 50 μg/mL of streptomycin (ScienCell #0503). HepG2 cells (ATCC #HB-8065) were cultured in RPMI 1640 medium containing 10% FBS. 4 × 10^6^ cells were seeded in 75 cm^2^ CELLSTAR tissue culture flasks (Greiner Bio One #658 175, Kremsmünster, Austria) and were incubated at 37 °C for 24 h. Then, the cultured cells were rinsed twice with 10 ml of cold Dulbecco’s phosphate-buffered saline without Ca^2+^ and Mg^2+^ (DPBS) and detached by cell scraping into 10 ml of cold DPBS. Next, cells were pelleted in microcentrifuge tubes (Crystalgen #23-2051, Commack, NY, USA) by centrifugation at 300× *g* for 5 min at room temperature and resuspended with 200 to 600 µL of cold RIPA Lysis Buffer (Thermofisher #89900, Waltham, MA, USA) supplemented with Halt protease and phosphatase inhibitor cocktail (Thermofisher #78442), 5 mM EDTA, 1 mM PMSF (Thermofisher #36978) and 1 µM Pepstatin A (Thermofisher #78436). Cells were incubated on ice for 5 min with gentle mixing by inverting the microcentrifuge tubes every 30 s. Afterwards, the cell lysates were centrifuged 18,000× *g* at 4 °C for 15 min to pellet cell debris. The supernatants were transferred to new tubes and protein concentrations were measured by the bicinchoninic acid method (Thermofisher #23227) using bovine serum albumin (BSA) as a standard.

Pre-stained molecular weight markers (BioDynamics Laboratory #DM660, Columbus, OH, USA) and 20 µg of cell lysate protein supernatant were loaded onto 4 to 12% NuPAGE Bis-Tris gradient gels (Invitrogen #NP0341, Carlsbad, CA, USA). Electrophoresis was carried out at constant 200 V for 45 min using the XCell SureLock mini-cell electrophoresis system (Invitrogen # EI000) in NuPAGE MES SDS running buffer (Invitrogen # NP0002) with a PowerPack Basic Power Supply (Bio-Rad Laboratories #1645050, Hercules, CA, USA). The gels were then transferred at 4 °C to nitrocellulose membranes using a Mini Trans-Blot electrophoretic transfer cell (Bio-Rad Laboratories #1703930) in NuPAGE Transfer Buffer (25 mM Bicine, 25 mM Bis-tris, 1 mM ethylenediaminetetraacetic acid, 0.05 mM chlorobutanol, 20% methanol, pH 7.2) (Invitrogen # NP0006) with a PowerPack Basic Power Supply. Next, the membranes were blocked for 1 h at room temperature with DPBS containing 5% non-fat dry milk and 0.05% Tween-20 (Bio-Rad Laboratories #170-6531). After blocking, the nitrocellulose membranes were immunoblotted overnight at 4 °C with 0.4 µg/mL rabbit monoclonal anti-ApoA1 (Abcam #ab52945, Cambridge, United Kingdom), 3.5 µg/mL rabbit monoclonal anti-LCAT (Abcam #ab109417), 1.0 µg/mL rabbit polyclonal anti-ApoD (Thermofisher #PA5-27386), or 1 µg/mL mouse monoclonal anti-β-Actin (Invitrogen #MA5-15739) diluted in DPBS containing 1% non-fat dry milk and 0.05% Tween-20. After washing three times with DPBS containing 0.05% Tween-20, the membranes were incubated at room temperature for 1 h with either horseradish peroxidase-conjugated donkey anti-rabbit IgG secondary antibody (GE Healthcare #NA934V, Chicago, IL, USA) or horseradish peroxidase-conjugated sheep anti-mouse IgG secondary antibody (GE Healthcare #NA931V) as specified by the manufacturer. Horseradish peroxidase was detected using enhanced chemiluminescence (Thermofisher #34577).

### 2.2. RNA Sequencing Analysis

Human keratocytes were cultured, rinsed, and pelleted as described above and stored at −80 °C until use. RNA was extracted with TRIzol (Invitrogen #15596026, Carlsbad, CA, USA) and purified with the QIAGEN RNeasy mini kit (QIAGEN, Germantown, MD, # 74104, country) as directed by the manufacturer. Residual DNA was removed by on-column DNase treatment. The sequencing libraries were constructed from 100 to 500 ng of total RNA using Illumina’s (San Diego, CA, USA) TruSeq Stranded Total RNA kit with Ribo-Zero following the manufacturer’s instructions. The fragment size of RNAseq libraries was verified using the Agilent 2100 Bioanalyzer (Santa Clara, CA) and the concentrations were determined using a Qubit instrument (LifeTech, Waltham, MA, USA). The libraries were loaded onto the Illumina HiSeq 2000 for 2 × 50 bp paired-end read sequencing. The fastq files were generated using the Illumina bcl2fastq software for further analysis. Raw reads were pre-processed with Cutadapt for adapter contamination [27]. Reads were aligned to the reference genome (GRCh38) using STAR (version 2.5.3a) [28]. Gene counts were generated by using featureCounts [29]. Fragments per kilobase of transcript per million mapped reads (FPMK) were calculated for each gene from triplicate keratocyte culture samples. 

### 2.3. Immunofluorescence Analysis

In the case of cultured cells, 1 × 10^5^ cells/well were seeded in Corning CellBIND 24 multi-well plates (Sigma #CLS3337, St. Louis, MO, USA) and 24 h later were rinsed two times with DPBS with Ca^2+^ and Mg^2+^ followed by fixation at room temperature for 10 min with DPBS containing 4% paraformaldehyde and subsequently permeabilized with 0.1% Triton-X in DPBS for an additional 10 min. After rinsing with DPBS with Ca^2+^ and Mg^2+^ three times, the cells were blocked for 1 h at room temperature with DPBS with Ca^2+^ and Mg^2+^ containing 1% BSA (essentially fatty acid and globulin free) (Sigma #A0281). After blocking, the cells were incubated at 4 °C overnight with 10 µg/mL rabbit monoclonal anti-ApoA1 (Abcam #ab52945), 0.78 µg/mL rabbit monoclonal anti-LCAT (Abcam #ab51060) or 1.0 µg/mL goat polyclonal anti-ApoD (Novus #NBP2-42526, Centennial, CO) diluted in DPBS with Ca^2+^ and Mg^2+^ containing 1% BSA. After rinsing three times with DPBS with Ca^2+^ and Mg^2+^, cells were incubated for 1 h at room temperature with either 2 µg/mL Alexa Fluor 488 chicken anti-rabbit IgG secondary antibody (Invitrogen #A21441) or 2 µg/mL Alexa Fluor 488 chicken anti-goat IgG secondary antibody (Invitrogen #A21467) diluted in DPBS with Ca^2+^ and Mg^2+^ containing 1% BSA. Mounting and nuclear counterstaining were done with VECTASHIELD Hard-Set mounting media containing 4′,6-diamidino-2-phenylindole (DAPI) (Vector Laboratories # H-1500, Burlingame, CA, USA). Cells were imaged using an Olympus 20×/0.45 numeric aperture objective and IX81 conventional fluorescence microscope (Center Valley, PA, USA). DNA blue fluorescence was imaged using 350/50 nm excitation and 460/50 nm emission filters. ApoA1, ApoD, and LCAT green fluorescence was imaged using 480/40 nm excitation and 535/50 nm emission filters. IPLab software (Scanalytics, Fairfax, VA, USA) was used to acquire and pseudocolor the digital images. 

In the case of tissue, the following procedures were followed. Normal human corneas from 3 donors were obtained from the Lions Eye Bank (Tampa, FL, USA). Corneas were maintained in Optisol-GS corneal storage medium (Bausch & Lomb Incorporated, Rochester, NY, USA) for a maximum of 72 h. Upon arrival, corneas were snap frozen in OCT medium, sectioned at 7 to 10 µm and mounted on slides. The sections were stored at −80 °C. For immunostaining, frozen sections were thawed for 30 min at room temperature and except for LCAT immunostaining, were fixed for 10 min at room temperature in 20 mM Tris-buffered saline (pH 7.4) (TBS) (Corning #46-012-CM, Manassas, VA, USA) containing 4% paraformaldehyde. Tissue sections immunostained with rabbit monoclonal anti-LCAT were not fixed with paraformaldehyde because fixation resulted in loss of antigenicity. After rinsing three times with TBS, the tissue sections were blocked for 2 h at room temperature with 1% BSA dissolved in TBS. After blocking, the tissue sections were immunostained overnight at 4 °C with 10 µg/mL rabbit monoclonal anti-ApoA1 (Abcam #ab52945), 0.8 µg/mL rabbit monoclonal anti-LCAT (Abcam #ab51060) or 1 µg/mL goat polyclonal anti-ApoD (Novus #NBP2-42526, Centennial, CO, USA) diluted in DPBS with Ca^2+^ and Mg^2+^ containing 1% BSA. Goat IgG isotype-matched (Invitrogen #02-6202) and monoclonal rabbit IgG isotype-matched antibody (Abcam #ab172730) served as negative controls. After overnight incubation, the tissue sections were rinsed three times with TBS, then immunostained for 1 h at room temperature with either 2 µg/mL Alexa Fluor 488 chicken anti-rabbit IgG secondary antibody (Invitrogen #A21441) or 2 µg/mL Alexa Fluor 488 chicken anti-goat IgG secondary antibody (Invitrogen #A21467) diluted in DPBS with Ca^2+^ and Mg^2+^ containing 1% BSA. Mounting, counterstaining, image acquisition, and processing was carried out as described above for cells. 

### 2.4. Lipid Analysis of Corneas

Cornea tissue removed during corneal transplant from a 55-year-old woman with fish-eye disease (no renal disease) [30], and cornea tissue obtained during autopsy from a 72-year-old man with Tangier disease [31,32] were analyzed for cholesterol and phospholipid content as previously described [33]. In brief, corneal tissue was weighed and extracted with a chloroform-methanol mixture according to Folch et al. [34]. Unesterified and esterified cholesterol was assayed with an enzymatic-fluorometric method [35], while phospholipid was determined by a colorimetric method [36]. Data reported for human tissue is exempt from IRB review in accordance with exemption 45 CFR 46.101(b)(4) (for existing data, documents, records, and specimens).

### 2.5. Electron Microscopy

Portions of cornea tissue obtained at autopsy from a 45-year-old woman with ApoA1 deficiency (later determined to be a deficiency of ApoA1, ApoC3, and ApoA4) [37] and from the Tangier patient described above were prepared for electron microscopy using the sequential tannic acid and p-phenylenediamine treatments of glutaraldehyde-fixed and osmicated tissue as described by Guyton and Klemp [38]. While this treatment helps preserve lipid, there was no ultrastructural difference from when this corneal tissue was processed for electron microscopy in the conventional manner as previously described [30] and as follows briefly here. After fixation in 4% glutaraldehyde in 0.1 M phosphate buffer (pH 7.2) at room temperature for 1 h, tissue was post-fixed for 2 h in 1% osmium tetroxide, dehydrated in ascending concentrations of ethanol, and embedded in Epon. After polymerization of Epon, tissue was sectioned for electron microscopy.

### 2.6. In Situ Hybridization

In situ hybridization detection of LCAT, ApoD, and ApoA1 mRNA in frozen sections of cornea was carried out using the RNAscope methodology [39] as described by the manufacturer (Advanced Cell Diagnostics, Newark, CA, USA). In brief, tissue sections are fixed onto slides for 1 h at 4 °C with 4% paraformaldehyde in phosphate-buffered saline (pH 7.4) and then treated to unmask RNA. Next, sections were incubated with target-specific probe pairs (Advanced Cell Diagnostics #s 577541, 445171, and 314311 for LCAT, ApoD, and ApoA1, respectively) that hybridize to the target RNA. Furthermore, a negative control was employed, a probe targeting the dihydrodipicolinate reductase gene from the *Bacillus subtilis* strain SMY (Advanced Cell Diagnostics # 310043). Hybridization signals were amplified and detected using alkaline phosphatase and Fast Red chromogenic detection. The method can detect a single mRNA molecule. Brightfield photomicrographs were taken with the Olympus microscope described above.

## 3. Results

**Figure 2:**
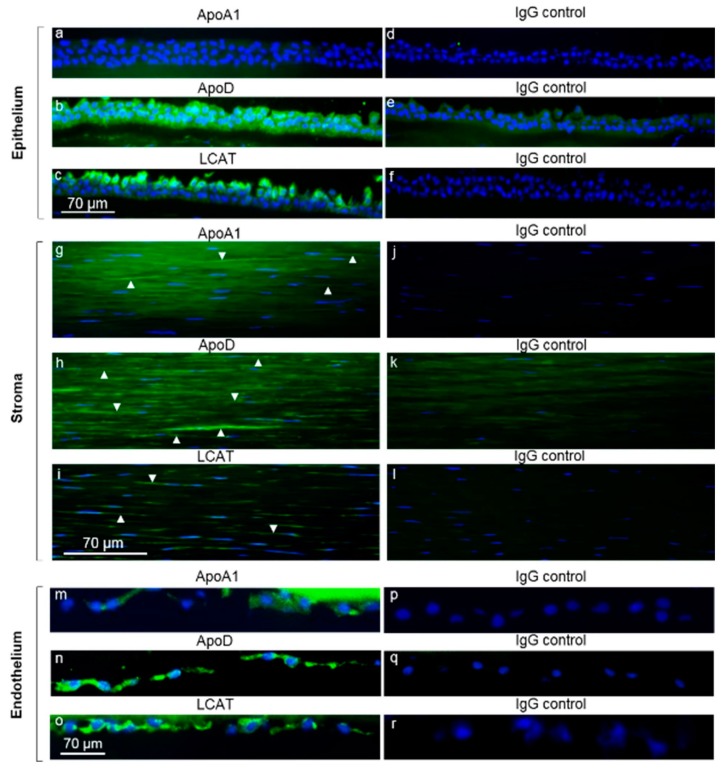
Immunostaining of apolipoprotein (Apo)A1, ApoD, and lecithin:cholesterol acyltransferase (LCAT) in the human central cornea. Frozen sections from an individual cornea were incubated at 4 °C with either anti-ApoA1 (**a**,**g**,**m**), ApoD (**b**,**h**,**n**) or LCAT (**c**,**i**,**o**) antibodies (green). Nuclei were stained blue with DAPI. Control sections (right panel) were incubated with the same concentrations of either rabbit IgG (**d**,**f**,**j**,**l**,**p**,**r**) or goat IgG (**e**,**k**,**q**). Antibodies were detected as described in the Materials and Methods. The epithelium, stromal keratocytes, and endothelium showed staining of ApoD (**b**,**h**,**n**) and LCAT (**c**,**i**,**o**). ApoA1 was detected in the stromal keratocytes and endothelium (**g**,**m**), but not in the epithelium. Arrowheads indicate some of the immunolabeled keratocytes.

Because patients with LCAT deficiency show accumulation of cholesterol that results in corneal clouding, we immunolabeled human cornea to learn whether LCAT and a putative activator, ApoD, were present in cornea (Figure 2). Because patients with ApoA1 deficiency also show corneal clouding, we also immunolabeled ApoA1 in the cornea. Both LCAT and ApoD proteins were localized within corneal epithelium, keratocytes, and endothelium (Figure 2b,c,h,i,n,o). ApoD was present throughout the epithelium (Figure 2b), while LCAT was present within the more superficial epithelial cells (Figure 2c). In contrast to ApoD and LCAT, which were present within all 3 cell types within the cornea, ApoA1 was present in both keratocytes and endothelium but was absent from the epithelium (Figure 2a,g,m). While both ApoD and ApoA1 were also present in the stromal extracellular matrix (Figure 2g,h), LCAT was not detected in the extracellular matrix (Figure 2i). Findings in the peripheral cornea limbus region were similar to the findings in the more central corneal region (Supplemental Figure S1). There was no specific staining when control isotype-matching IgG was substituted for the specific antibodies (Figure 2d,e,f,j,k,l,p,q,r).

**Figure 3:**
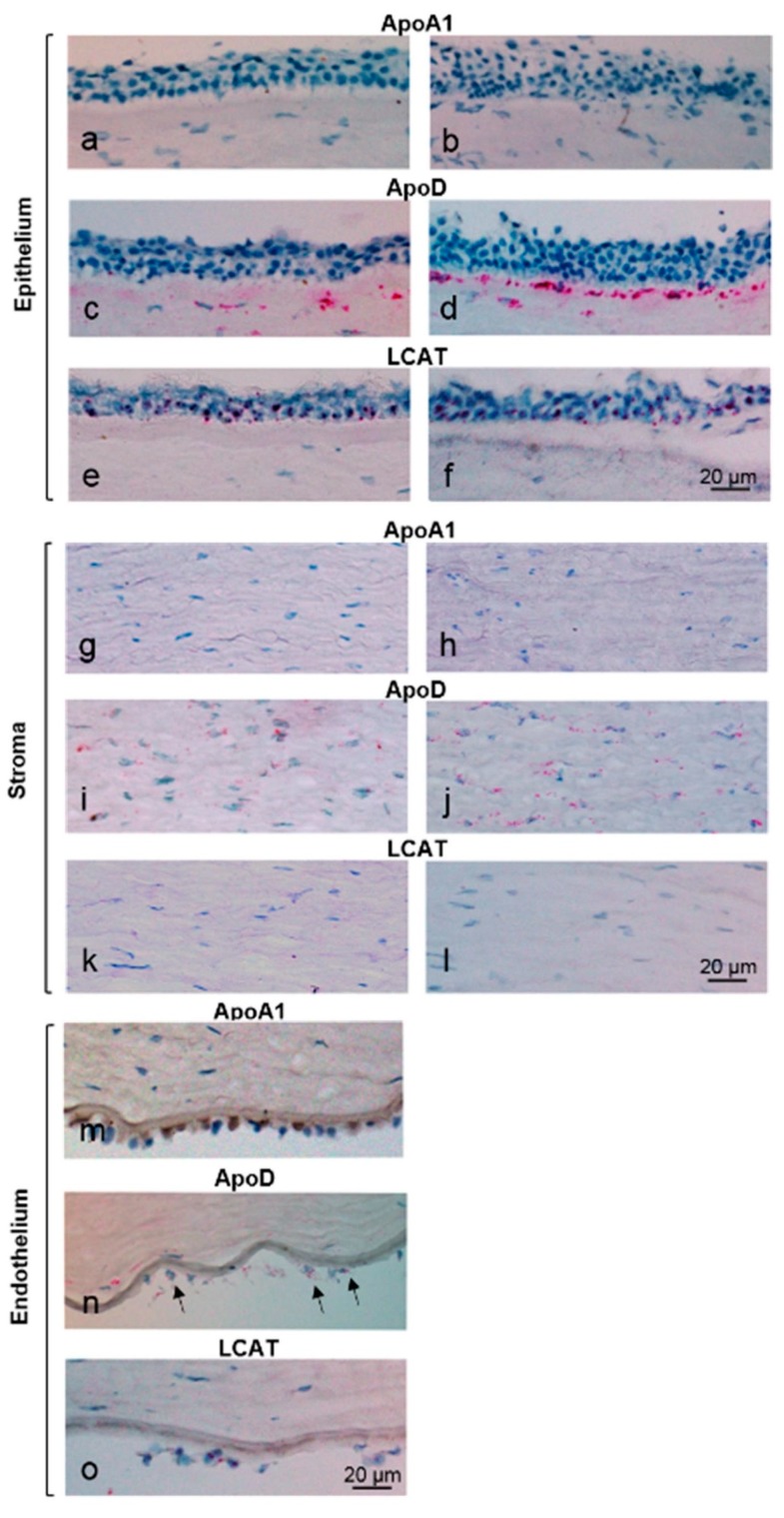
In situ hybridization of ApoA1, ApoD, and LCAT mRNAs in the human cornea. The left-hand column shows the central cornea and the right-hand column shows the peripheral limbus region of the cornea. The in situ hybridization signal consists of red dots present within the cells. (In the endothelium, they are best seen after screen enlargement of the images.) Note, there are no endothelial cells in the limbus region of the cornea. ApoA1 mRNA was not detected in any corneal cells (**a**,**b**,**g**,**h**,**m**). ApoD mRNA was absent from the epithelium (**c**,**d**) but was present within stromal keratocytes (**i**,**j**) with greatest amount of mRNA within anterior stromal keratocytes (lower portion of (**c**,**d**). Endothelium (**n**) also showed ApoD mRNA (arrows indicate endothelial cells artifactually separated from the cornea). LCAT mRNA was present within epithelial cells (**e**,**f**) and endothelial cells (**o**) but was absent from keratocytes (**k**,**l**).

To learn whether the immunolocalized proteins were synthesized by the different corneal cell types, we carried out in situ hybridization analysis to detect the presence of each protein’s corresponding mRNA. Despite ApoA1 immunolocalization to cornea endothelium and keratocytes, there was no mRNA for this protein (Figure 3a,b,g,h,m). Likewise, although keratocytes showed immunolocalization of LCAT protein, LCAT mRNA was not present in the keratocytes (Figure 3k,l). On the other hand, LCAT mRNA was present in endothelium and epithelium (Figure 3e,f,o), which also showed LCAT protein as noted above. ApoD mRNA was present in keratocytes and endothelium (Figure 3i,j,n) but was absent from epithelium (Figure 3c,d). ApoD mRNA was most prominent in keratocytes in the anterior stroma just below the epithelium (Figure 3c,d).

**Figure 4:**
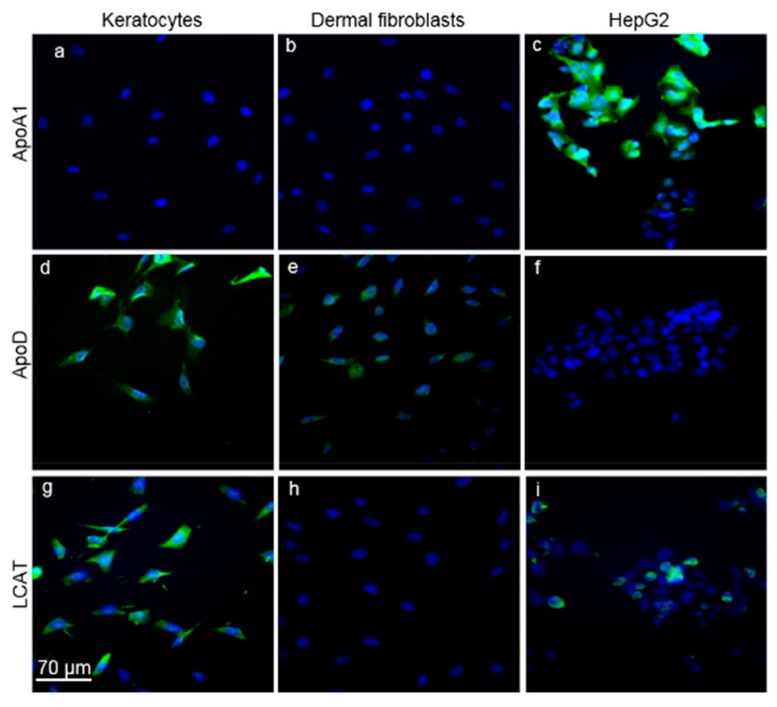
ApoA1, ApoD, and LCAT immunolabeling in cultured keratocytes, dermal fibroblasts, and HepG2 liver cells. Cells were immunolabeled with either anti-ApoA1 (**a**–**c**), ApoD (**d**–**f**) or LCAT (**g**–**i**) antibodies (green). DAPI was used to visualize DNA (blue). Anti-ApoA1 only immunolabeled HepG2 positive control cells (**c**), whereas anti-LCAT immunolabeled both HepG2 positive control cells (**i**) and keratocytes (**g**), but not dermal fibroblasts (**h**). Anti-ApoD immunolabeled keratocytes (**d**) and dermal fibroblasts (**e**), but not HepG2 cells (**f**).

Next, we determined if ApoA1, ApoD, and LCAT proteins were expressed in cultured keratocytes (Figure 4). The anti-ApoA1 antibody labeled the cytosol of cultured HepG2 cells, a hepatocyte cell line that served as a positive control (Figure 4c). However, despite anti-ApoA1 immunolabeling of keratocytes in vivo, the anti-ApoA1 did not label cultured keratocytes (Figure 4a) or cultured dermal fibroblasts that served as a negative control (Figure 4b). Anti-ApoD antibody labeled the cytosol of keratocytes (Figure 4d) and dermal fibroblasts (Figure 4e) but did not label HepG2 cells (Figure 4f). Anti-LCAT antibody labeled keratocytes (Figure 4g) and as expected labeled HepG2 positive control cells (Figure 4i) but not negative control dermal fibroblasts (Figure 4h).

**Figure 5:**
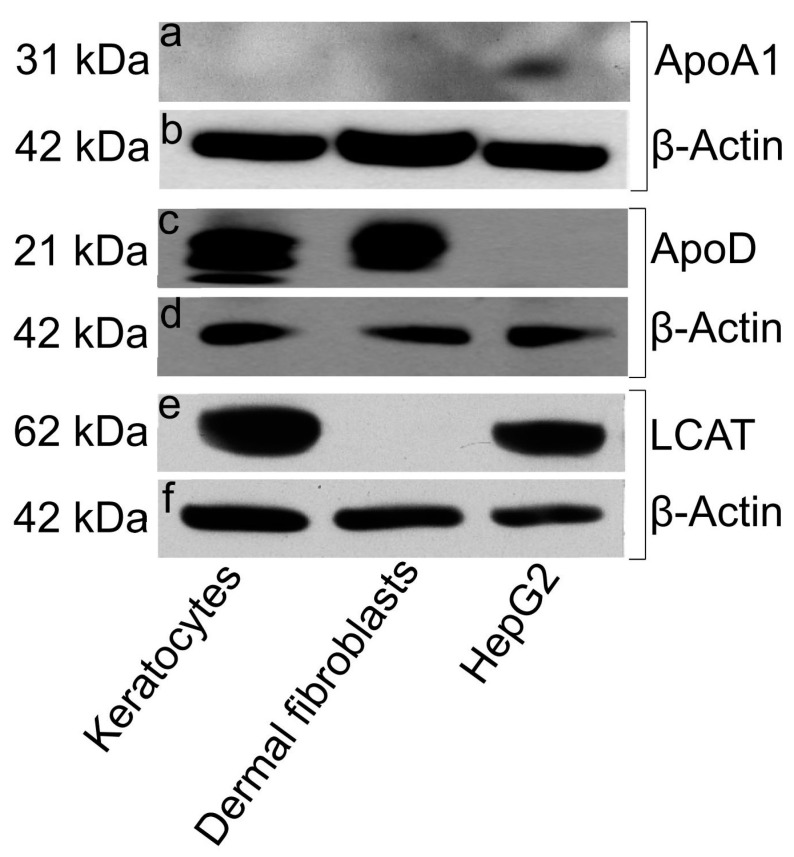
ApoD and LCAT proteins, but not ApoA1 protein, were present in keratocytes. In a Western blot assay, lysates from keratocytes, dermal fibroblasts and HepG2 cells were solubilized and loaded onto 4 to 12% NuPAGE Bis-Tris gradient gels. After electrophoresis, resolved proteins were transferred onto a nitrocellulose membrane and incubated with antibodies against either ApoA1 (**a**), ApoD (**c**) or LCAT (**e**). Anti-β-Actin served as a loading control (**b**,**d**,**f**).

To confirm our immunostaining findings, we carried out Western blot analysis of cell lysates for the three cell types probing for ApoA1, ApoD, and LCAT proteins with antibodies to each (Figure 5). ApoD and LCAT were detected at high levels in keratocytes (Figure 5c and e), while ApoD was detected at similar levels in dermal fibroblasts, but not in negative control HepG2 cells (Figure 5c). ApoA1 was only detectable in HepG2 positive control cells but was not detected in keratocytes or dermal fibroblasts (Figure 5a). These observations demonstrate that ApoD and LCAT are present in cultured keratocytes, whereas ApoA1 is not.

Given that both immunostaining and Western blot analysis showed the presence of LCAT and ApoD protein within cultured keratocytes, we carried out RNA sequencing analysis (Table 1) to learn whether the cultured keratocytes expressed mRNA for these proteins indicating the proteins were synthesized by the cultured keratocytes, rather than possibly originating from the culture medium. This analysis showed that the cultured keratocytes expressed both LCAT and ApoD as well as other proteins that potentially function in reverse cholesterol transport from the cornea (e.g., ABCA1, ApoE, PLTP, ApoC1, SCARB1). No mRNA for ApoA1 was detected consistent with the negative immunostaining result for this apolipoprotein. The keratocyte differentiation markers keratocan and aldehyde dehydrogenase 1 were not expressed by the cultured keratocytes.

**Figure 6:**
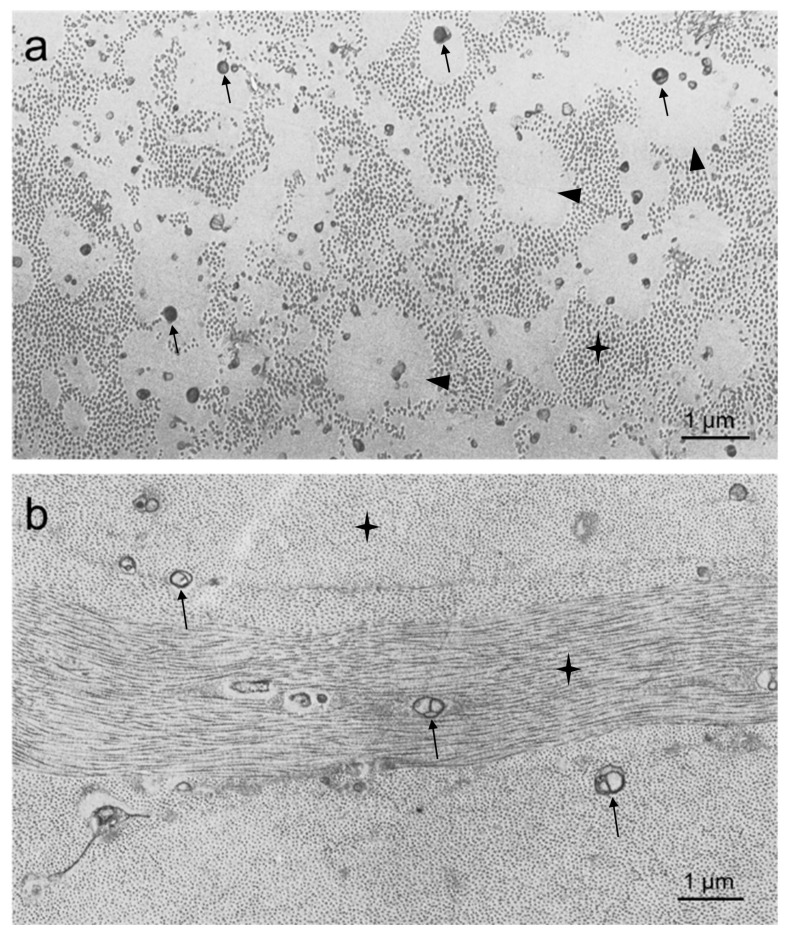
Electron microscopy of deposits in genetic deficiency of ApoA1 (**a**) and ABCA1 (Tangier disease) (**b**). Note that consistent with clinical appearance, the stromal deposits in ApoA1 deficiency are more extensive than the stromal deposits in ABCA1 deficiency. In ApoA1 deficiency, there is a large amount of amorphous component (arrowheads) along with the membranous component that predominates in ABCA1 deficiency (arrows). Collagen fibers (indicated by star) appear on edge in (**a**), and both on edge and in parallel in (**b**).

As discussed above, both ApoA1 and ABCA1 deficiencies result in central corneal clouding like LCAT deficiency, but the severity of clouding is much greater in LCAT and ApoA1 deficiency compared with ABCA1 deficiency (Table 2). We report here the first electron microscopic examination of a cornea from an individual with ApoA1 deficiency (Figure 6a) and compare it with a cornea from an individual with ABCA1 deficiency (Figure 6b). Like previously reported for LCAT deficiency [30,40,41,42,43], the ApoA1-deficient cornea showed embedded among the stromal collagen fibrils large 0.5–2 µm round and oval spaces filled with an amorphous material and generally one to five membranous inclusions (Figure 6a). Keratocytes appeared normal and did not show lipid accumulation. Although the patient reported here with ApoA1 deficiency also had genetic deficiency of ApoC3 and ApoA4, corneal clouding due to lipid deposition was most likely due to the ApoA1 deficiency. This is because individuals with isolated genetic ApoA1 deficiency also show corneal clouding [4]. In contrast to the ApoA1-deficient cornea, the ABCA1-deficient cornea showed only sparse membranous structures and lacked the amorphous deposits observed in the ApoA1-deficient cornea. Keratocytes appeared normal without lipid accumulation.

We also report here cholesterol and phospholipid accumulation in a cornea from a patient with ABCA1 deficiency and LCAT deficiency (Fish-eye disease) (Table 3). The amount of lipid accumulation paralleled the degree of corneal clouding observed in these two deficiencies. There was a 3-fold increase in unesterified cholesterol in the ABCA1-deficient cornea relative to normal corneas, whereas there was a 19-fold increase in unesterified cholesterol in the LCAT-deficient cornea. There was no increase in esterified cholesterol in either cornea compared to normal corneas. Phospholipid content did not increase in the ABCA1-deficient cornea but increased 7.5-fold in the LCAT deficient cornea. The unesterified cholesterol to phospholipid molar ratio for both diseased corneas increased to 1.3 compared with 0.5 for control corneas.

## 4. Discussion

### 4.1. Corneal Expression of LCAT, ApoD, and ApoA1

Here we have shown that LCAT protein is present in the cornea, in stromal keratocytes, epithelium, and endothelium. While both epithelium and endothelium showed the presence of LCAT mRNA indicating their content of LCAT was due to synthesis by these cells, stromal keratocytes lacked mRNA suggesting that their content of LCAT resulted from uptake from the corneal interstitial tissue fluid. Despite a lack of LCAT mRNA in keratocytes in vivo, RNA sequencing analysis showed a low level of LCAT mRNA that could explain the presence of LCAT protein in cultured keratocytes. It is well known that keratocytes dedifferentiate and transform to corneal stromal fibroblasts when cultured in serum, losing the differentiation markers keratocan and aldehyde dehydrogenase 1 as was the case in our study [45]. With this dedifferentiation, our findings indicate that cultured keratocytes gain a low level of LCAT expression. LCAT expression in the corneal epithelium resembles its expression in the skin epithelium [11] where its function is unknown. While one might suppose that LCAT in the epithelium esterifies cholesterol there, LCAT- deficient patients have not been reported to have any skin or corneal epithelial-related symptoms. Deficiency of LCAT is associated with corneal clouding that is due to deposition of cholesterol and phospholipid (Table 3) [30,40,41,42,43,46] throughout the extracellular matrix of the corneal stroma. Thus, corneal expression of LCAT may function to facilitate reverse cholesterol transport from the cornea, preventing lipid accumulation and resultant impairment of vision due to corneal clouding. Although LCAT is secreted from the liver and circulates bound to plasma HDL [47,48], local expression of LCAT in the cornea either constitutes the major source of cornea LCAT or functions to supplement LCAT that enters the cornea from the circulation. 

ApoD expression by keratocytes and endothelial cells could facilitate activation of LCAT. ApoD could also function in the cornea as a cholesterol transport protein but cholesterol binding to ApoD is of low affinity and its physiological relevance is not clear [49,50]. We also observed ApoD protein within the epithelial cell layers that overlie the corneal stroma. However, the epithelium lacked mRNA for ApoD indicating that the ApoD is produced exogenously. It is likely the source of this ApoD is from the lacrimal gland which is known to synthesize ApoD [51]. The corneal epithelium is covered by a layer of mucin and a lipid film secreted by the meibomian glands [52]. The function of these layers is to maintain a suitable surface tension that facilitates wetting of the cornea surface. Tear lipocalins, including ApoD [53], produced by the lacrimal gland bind various lipids and help maintain an optimal cornea surface tension through their binding to cornea surface lipids [54,55].

We previously observed and have confirmed in this study that ApoA1 besides being present within the corneal stroma extracellular matrix also was present within cornea keratocytes and endothelial cells [23]. We have shown here that this intracellular pool of ApoA1 is not due to cellular synthesis because the cells lacked any ApoA1 mRNA. Various cells are known to take up HDL without subsequently degrading it, but rather the HDL is resecreted [56]. Furthermore, ApoA1 presence has been reported within vascular smooth muscle cells, adventitial fibroblasts, and renal cell carcinoma cells [57,58,59,60], cell types that likely do not synthesize ApoA1, suggesting that some cells can take up and accumulate ApoA1. ApoA1′s absence within cultured keratocytes in contrast to in vivo keratocytes suggests that the ApoA1 is taken up by keratocytes in vivo but that this does not occur when keratocytes are cultured. 

### 4.2. Morphology of Corneal Lipid Deposits

Electron microscopic studies of corneas from individuals with genetic LCAT deficiencies and for the first time as reported here of a cornea from an individual with familial ApoA1 deficiency [30,40,41,42,43] show rather similar findings. In both cases, the corneal stroma shows extracellular round to oval vacuolar spaces varying in size from 0.2 to 2.5 µm in diameter, and much larger irregularly shaped extracellular spaces that appear to form from coalescence of smaller vacuoles. The vacuolar spaces are embedded between collagen fibrils and show regions that appear empty or contain membranous and amorphous material. Generally, the cells of the cornea appear normal except for some signs of degeneration in a few of them. Ultrastructurally, the lipid in Schnyder corneal dystrophy (defective UBIAD1) also accumulates as extracellular membranous and vacuolar structures like that seen in LCAT deficiency, but additionally within cholesterol crystals in about half the cases [61,62,63]. In the case of LCAT deficiency and Schnyder corneal dystrophy, histochemical and chemical analysis including that presented here show the presence of cholesterol and phospholipid as a component of the extracellular lipid deposits. Corneas of Schnyder corneal dystrophy show accumulation of cholesterol, about 37% of which is esterified [33] in contrast to familial LCAT deficiency [46,64] and fish-eye disease (Table 3) [40], where most reports indicate that ≤15% of cholesterol is esterified. 

Recently, we have identified a previously unknown form of extracellular cholesterol–phospholipid complex that is shed from the plasma membrane and deposits into the extracellular matrix of cultured cholesterol-enriched macrophages [9]. These deposits also have an amorphous appearance suggesting the possibility that cornea keratocytes likewise shed or release into the extracellular space by some other means similar extracellular cholesterol–phospholipid complexes. Both ABCA1 and ABCG1 mediate macrophage shedding (and shedding by other cell types) of the extracellular cholesterol–phospholipid complexes [65,66]. With ABCA1 deficiency (i.e., Tangier disease), there is very mild corneal clouding (usually requiring a slit-lamp examination for its detection) and less abundant extracellular corneal stromal deposits, cholesterol, and phospholipid accumulation compared with genetic deficiency of LCAT (Table 3 and Figure 6). If cholesterol-phospholipid deposition in the cornea were due to shedding of these lipids from keratocytes, less abundant extracellular lipid deposits would be expected because of loss of the ABCA1-mediated component of the extracellular deposition of cholesterol-phospholipid. 

### 4.3. Source of Cholesterol that Accumulates in Cornea 

The source of the cholesterol that accumulates within the corneal stroma due to disorders of reverse cholesterol transport such as LCAT, ABCA1, and ApoA1 genetic deficiencies remains to be determined. LDL’s 22 nm size precludes its filtration into the central cornea because the largest particle size capable of diffusing throughout the central stromal matrix is about 12 nm [24]. On the other hand, normal plasma HDL is small enough (7–11 nm diameter) [67] to enter and diffuse throughout the corneal stroma. Consistent with this is the finding that ApoA1 but not ApoB, major protein components of HDL and LDL, respectively, localizes throughout the normal corneal stroma [21,22,23,33]. In contrast, ApoB is present only in the periphery of the corneal stroma. 

Lipoprotein X is an abnormal unesterified cholesterol-phospholipid containing liposome-like lipoprotein that accumulates in the plasma of patients with familial LCAT deficiency [68,69,70,71]. There is evidence that lipoprotein X accumulates within the kidney and causes the renal disease that occurs in patients with familial LCAT deficiency [72,73]. However, it is unlikely that lipoprotein X is the source of the lipid deposits in the cornea. As discussed above for LDL, the size of lipoprotein X, 34 to 120 nm in diameter [68,70], would preclude its passage into the central cornea. Moreover, lipoprotein X is usually absent in fish-eye disease [70,74], a partial LCAT deficiency, yet patients with both fish-eye disease and familial LCAT deficiency develop cloudy corneas due to corneal lipid accumulation.

Besides lipoprotein X, abnormal HDL lipoproteins accumulate within the plasma of patients with LCAT deficiency. These abnormal HDL particles include nascent discoidal HDL that are normally transformed to mature spherical HDL when there is enough LCAT to convert their unesterified cholesterol to cholesteryl ester [14,75]. The majority of these discoidal HDL are 15–20 nm in their largest diameter dimension [68], which like lipoprotein X and LDL may be too large to penetrate into the central cornea. However, small to normal-sized spherical HDL particles are found in very small amounts in familial LCAT deficiency, fish-eye disease, and Tangier disease [68,74,76] and these could transport cholesterol into the cornea. 

Cholesterol synthesized within the cornea [77] is another possible source of cholesterol that accumulates there when ABCA1, ApoA1 and LCAT mediators of reverse cholesterol transport are deficient. Consistent with cholesterol synthesis as a source of accumulated cholesterol in the cornea, mutation of the *UBIAD1* gene causes Schnyder corneal dystrophy, which as discussed above results in corneal clouding due to accumulation of extracellular lipid deposits [33,78]. It has been proposed that cholesterol accumulation in Schnyder corneal dystrophy is linked to defective regulation of HMGCoA reductase, the rate limiting enzyme in cholesterol synthesis [79]. Schnyder corneal dystrophy mutant UBIAD1 interferes with sterol-induced HMGCoA reductase degradation resulting in elevated cholesterol synthesis in vitro. 

### 4.4. Cholesterol Removal from the Cornea

Regardless of whether cornea accumulated cholesterol is derived from blood lipoproteins or is synthesized within the cornea, the lipid deposits in ABCA1, ApoA1 and LCAT deficiencies could accumulate due to a failure in mechanisms that remove excess cholesterol from the cornea. As discussed above, nascent discoidal HDL is an effective lipoprotein complex that solubilizes cholesterol. Keratocyte ABCA1-mediated complexing of ApoA1 with phospholipid could generate this discoidal HDL in the cornea. While keratocyte generated discoidal HDL has not been studied, nascent discoidal HDL produced by other cell types generally range in size from 15 to 25 nm in diameter [80,81,82,83,84]. In the absence of LCAT, discoidal HDL generated in the cornea would likely be trapped in the cornea extracellular matrix because LCAT would not convert discoidal HDL into smaller spherical HDL. Over time, loss of stabilizing protein from extracellular cholesterol–phospholipid complexes could convert the lipid complexes into larger liposomes [85] resembling the membranous lipid material observed within the corneas.

Our findings indicate that keratocytes express ApoE and ApoC1 (Table 1), apolipoproteins that like ApoA1 generate nascent discoidal HDL through interaction with ABCA1, and which like ApoD can activate LCAT [15,16,26,86]. However, ApoA1 is the most effective activator of LCAT and only ApoA1 generates the smallest spherical HDL particles [75]. Previously, we showed that while both ApoE and ApoA1 cholesterol-carrying lipid particles could be isolated from human cornea, gel-filtration showed that the ApoA1 lipid particles were smaller than the ApoE lipid particles [22]. Just as size could be the rate-limiting factor for lipid particle entry into the cornea, size likely is a rate-limiting factor for lipid particle removal from the cornea. Thus, even with LCAT activation by other apolipoproteins, without ApoA1, reverse cholesterol transport from the cornea may not occur because HDL sufficiently small to efflux from the cornea may not be generated.

In conclusion, we have shown that LCAT and ApoD are expressed in the cornea. While ApoA1 is not expressed in the cornea, it accumulates within keratocytes. These proteins along with ABCA1 likely function to mediate reverse cholesterol transport from the cornea. Keeping excess cholesterol from building up in the cornea is critical to maintaining visual acuity and this may be the evolutionary reason for the cellular presence of these proteins within the cornea.

## Figures and Tables

**Table 1:** Gene expression assessed by RNA sequencing.

Gene	FPKM ± SD
*APOA1*	0
*APOA2*	0
*APOA4*	0
*APOC1*	2 ± 1
*APOC2*	0
*APOC3*	0
*APOD*	72 ± 6
*APOE*	12 ± 1
*ABCA1*	20 ± 0
*ABCG1*	0
*PLTP*	24 ± 4
*CETP*	0
*LCAT*	3 ± 1
*LIPC*	0
*LIPG*	0
*LPL*	0
*SCARB1*	7 ± 1

RNA sequencing was carried out on normal human cultured keratocytes. Results are means ± standard deviation for triplicate keratocyte samples. FPKM = fragments per kilobase of transcript per million mapped reads and is a measure of RNA expression for the indicated genes. SD = standard deviation.

**Table 2:** Effect of reverse cholesterol transport gene deficiencies on cornea and coronary artery disease.

Gene	Degree of Central Corneal Cloudiness	Peripheral Arcus	Effect on Coronary Artery Disease
*ABCA1*	mild	absent	mild increase
*APOA1*	moderate	present	severe increase
*LCAT*	severe	present	no consistent effect

**Table 3:** Lipid analysis of corneas.

Disease	Affected Gene	Lipid Content, µmol/g Tissue (Wet Weight)					
		TC	UC	EC	PL	UC/PL	% UC
Normal	-	2 ± 0.6	1 ± 0.3	1 ± 0.3	2 ± 0.3	0.5 ± 0.03	50 ± 5
SCD	*UBIAD1*	27 ± 10	16 ± 3	11 ± 5	11 ± 1	1.5 ± 0.2	63 ± 7
Tangier	*ABCA1*	3	3	0	2	1.3	100 *
Fish-eye	*LCAT*	20	19	1	15	1.3	97

Lipid was extracted from portions of cornea and assayed as described in the Materials and Methods. Means ± standard error for normal and Schnyder corneal dystrophy (SCD) corneas were published previously [33] and are included here for comparison. TC, total cholesterol; UC, unesterified cholesterol; EC, esterified cholesterol; PL, phospholipid; UC/PL, molar ratio of unesterified cholesterol to phospholipid; %UC, percentage of cholesterol that was unesterified. * In contrast to our finding, Winder et al. [44] found only 38% of cholesterol was unesterified.

## Supplementary Materials

The following are available online at https://www.mdpi.com/2218-273X/9/12/785/s1, Figure S1: Immunostaining of ApoA1, ApoD, and LCAT in the peripheral limbus region of the human cornea.

## Abbreviations

Apo	apolipoprotein
BSA	bovine serum albumin
DPBS	Dulbecco’s phosphate-buffered saline
FBS	fetal bovine serum
HDL	high-density lipoprotein
LCAT	lecithin:cholesterol acyltransferase
LDL	low-density lipoprotein
TBS	tris-buffered saline
