# Supplementary material for: LCAT, ApoD, and ApoA1 Expression and Review of Cholesterol Deposition in the Cornea
Source: Biomolecules. 2019 Nov 26;9(12):785. doi: 10.3390/biom9120785 (PMC6995527; doi:10.3390/biom9120785)
Supplement: Supplementary file 1 [file biomolecules-09-00785-s001.pdf]

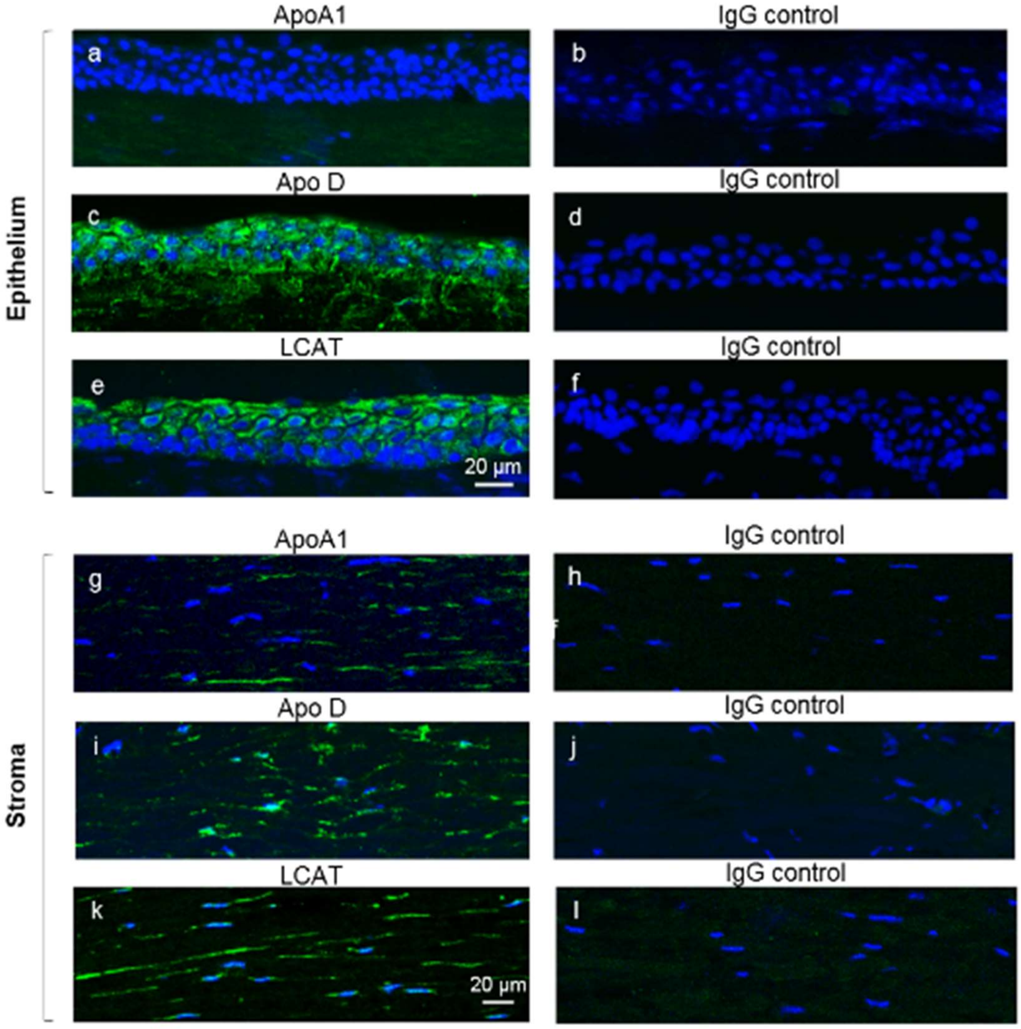

Figure S1: Immunostaining of ApoA1, ApoD and LCAT in the peripheral limbus region of the human cornea. Frozen sections from an individual cornea were incubated at 4°C with either anti-ApoA1 (a and g), ApoD (c and i) or LCAT (e and k) antibodies (green). Nuclei were stained blue with DAPI. Control sections (right panel) were incubated with the same concentrations of either rabbit IgG (b,f,h, and l) or goat IgG (d and j). Antibodies were detected as described in the Materials and Methods. Epithelium shows staining for ApoD and LCAT, while keratocytes show staining for all 3 proteins.
